# Sorghum Malt Extract as a Growth Medium for Lactic Acid Bacteria Cultures: A Case of *Lactobacillus plantarum* MNC 21

**DOI:** 10.1155/2020/6622207

**Published:** 2020-12-11

**Authors:** Stellah Byakika, Ivan Muzira Mukisa, Yusuf Byenkya Byaruhanga

**Affiliations:** Department of Food Technology and Nutrition, School of Food Technology Nutrition and Bioengineering, College of Agricultural and Environmental Sciences, Makerere University, P.O. Box 7062, Kampala, Uganda

## Abstract

Cultivation of lactic acid bacteria cultures is vital for research and commercial production of fermented foods. However, the conventional growth media used are generally costly. Malt extracts from four sorghum varieties (SESO 1, SESO 3, Epuripur, and Eyera) were evaluated as alternative low-cost growth media for *Lactobacillus plantarum* MNC 21. Saccharified sorghum malt extracts were inoculated with 4 log cfu/mL MNC 21 and incubated at 30°C for 24 h. MRS broth was the reference medium. Microbial counts, pH, titratable acidity (TA), free amino nitrogen (FAN), and total sugars were measured. Maximum microbial counts in the extracts and MRS broth were 9 and 10 log cfu/mL, respectively. Maximum growth rate in the extracts was 0.7–0.9 log cfu/mL/h and 0.8 log cfu/mL/h in MRS broth. The final pH of the extracts was 3.5–3.6, with an overall increase in TA of 1.2% in Epuripur and 0.2% in other varieties. Final pH and TA of MRS broth were 4.1 and 1.3%, respectively. Total sugars dropped by 95.2% and FAN by 2.1% in MRS broth. In contrast, total sugars and FAN dropped by 5.6–9.1% and 24.9–32.7% respectively, in the extracts. Sorghum malt extracts can be adopted as alternative low-cost growth media for lactic acid bacteria cultures.

## 1. Introduction

Lactic acid bacteria (LAB) are commonly applied as starter cultures for numerous fermented foods, cereals, tubers, fruits, vegetables, milk, fish, and meat. They impart characteristic flavors and tastes while contributing to food safety and preservation [1]. Some LAB strains such as *Lactobacillus* (*L.*) *rhamnosus* GG, *L. plantarum* 299v, and *L. casei* Shirota have probiotic properties such as alleviation of rotavirus diarrhea, prevention of traveller's diarrhea and *Clostridium difficile* colitis, prevention of atopic dermatitis, treatment of irritable bowel syndrome, increase in iron absorption, intestinal flora reposition, and improved digestion [2–5].

The growth of LAB is often restricted to rich-nutrient habitats, so they are cultivated in conventional media such as lactobacilli MRS broth, M17 broth, Micro Inoculum Broth, Rogosa SL Broth, Tomato Juice Broth, APT Broth, Elliker Broth, and *Lactobacillus* Selection Broth [6]. These media are costly owing to their composition and preparation, thus limiting their application to quality control, laboratory analysis, research, and academic uses [7]. So, it is important to develop alternative low-cost media for industrial applications like production of starter cultures and probiotics. Low-cost cultivation media from locally available materials such as mushrooms, tomatoes, corn, fish viscera hydrolysate, sweet potatoes, whey, buttermilk, pineapple peels, chick pea, cabbage, wheat, barley, and sugar beet molasses have been reported [8–18]. Sorghum is a low-cost cereal found in almost all parts of the world. It is mainly consumed as food and feed. Sorghum has not yet been exploited as a potential low-cost microbial cultivation medium, yet it can support growth of a diversity of LAB and yeasts in traditional fermentations [19, 20]. In its malted form, sorghum has a nutrient profile that could support the luxurious growth of LAB [21]. So, sorghum malt extract could be used for microbial cultivation, but this had not been evaluated previously.

*L. plantarum* are generally regarded as safe owing to their documented history of safe use [9, 22]. These LAB are thus widely used in food fermentation because of the sensory and shelf life qualities they render to food [22]. *L. plantarum* MNC 21, an isolate of Obushera, a fermented sorghum-millet beverage from Uganda, is a high acid producer and acid-stable microbe. It is thus a suitable starter culture for Obushera [23]. It is fastidious, and its successful cultivation in a given medium could indicate the same for several other microbes. Therefore, this study evaluated sorghum malt extracts as cultivation media for *L. plantarum* MNC 21.

## 2. Materials and Methods

### 2.1. Sorghum

Four improved sorghum varieties (SESO 1, SESO 3, Epuripur, and Eyera) were used in this study. SESO 1 and Epuripur are white-grained, whereas SESO 3 is brown-grained. Eyera is a popular local brown-grained variety. All four varieties were obtained from National Semi-Arid Resources Research Institute in Serere district, Uganda.

### 2.2. Sorghum Malt Extracts

Sorghum grain was malted following procedures described by Taylor [24]. The malted grain was milled using a Wonder Mill (110 Volt model, California, USA) and sieved using a 800 *μ*m screen. The flour was mixed with water to form a mixture of 11% total solids. To convert starch to maltose, the mixture was heated to 75°C, followed by addition of *α*-amylase (Anke Bio Engineering Company Limited, China) at a rate of 1000 units per mL. The slurry was held at 75°C for 1 h with continuous stirring. To convert maltose to glucose, the slurry temperature was lowered to 55°C, and amyloglucosidase (Anke Bio Engineering Company Limited, China) was added at a rate of 1000 units per mL. The slurry was held at 55°C for 1 h with continuous stirring. The malt extract was cooled to about 25°C, decanted, and filtered using grade filter papers (Whatman No. 1). It was then sterilized at 121°C for 15 min and cooled to 25°C.

### 2.3. Microbial Culture

*L. plantarum* MNC 21 isolated from Obushera by Mukisa [23] was used. From the stock culture, 0.1 mL was delivered into 100 mL of sterile MRS broth (CONDA, Madrid, Spain) and incubated at 30˚C for 24 h. *L. plantarum* MNC 21 was subcultured thrice after which the cells were recovered by centrifugation at 7,500 x g for 10 min. The cell pellets were suspended in 10 mL of sterile Ringer's solution. Culture purity was checked using a microscope (020–518.500 DM/LS I/98 model, Leica, Germany).

### 2.4. Propagation of L. *plantarum* MNC 21 in Sorghum Malt Extract

A hundred milliliters of sterile sorghum malt extract were inoculated with about 4 log cfu/mL of *L. plantarum* MNC 21 and incubated at 30°C for 24 h. Growth characteristics of MNC 21 were evaluated by determining microbial counts, pH, titratable acidity (TA), total sugars, and free amino nitrogen (FAN) at 2 h intervals for 24 h. MRS broth was also inoculated with MNC 21 and used as the control medium. The maximum growth rate (*μ*_max_) was calculated as described by [25].

### 2.5. Analyses

Microbial counts were determined by pour plating selected serial dilutions of the malt extract or MRS broth containing *L. plantarum* MNC 21 in MRS agar and incubating at 30°C for 48 h. The pH was determined using a pH meter (AG model, Mettler-Toledo Group, Switzerland). Titratable acidity was determined by titrating 10 mL of the extract against 0.1 N NaOH using phenolphthalein indicator [26]. FAN was determined using the Ninhydrin method [27]. Total sugars were determined using the Phenol-Sulfuric acid method [28].

### 2.6. Statistical Analyses

Results were presented as means ± standard deviations (mean ± SD) of three independent experiments. Data were subjected to one-way analysis of variance (ANOVA) to test for significant differences at *α* = 0.05. Mean comparisons were made using the least significant difference (LSD) test. Analyses were done using Statistix (student version 9.0) software.

## 3. Results and Discussion

### 3.1. Growth Characteristics of *L. plantarum* MNC 21

**Figure 1:**
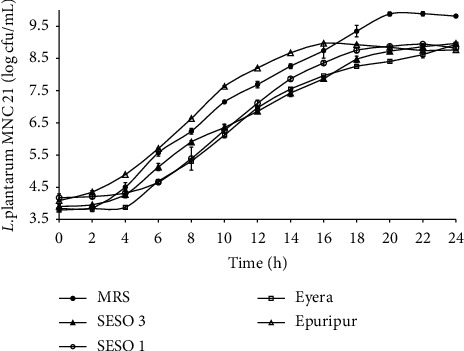
Growth profiles of *L. plantarum* MNC 21 in MRS broth and malt extracts from different sorghum varieties. Error bars show standard deviations of three independent fermentations.

Figure 1 and Table 1 summarize the growth characteristics of *L. plantarum* MNC 21 in MRS broth and malt extracts from different sorghum varieties. The LAB grew in all varieties, reaching a maximum average microbial count of 8.9 ± 0.0 log cfu/mL, one log cycle lower than what was observed in MRS broth. A steep increase in microbial counts in all media occurred in the first 4–18 h of fermentation and then plateaued. Maximum microbial counts were obtained earliest (16 h) in Epuripur and latest in MRS broth (22 h). Maximum growth rate (*μ*_max_) was in the order Epuripur > SESO 1 = MRS broth > SESO 3 = Eyera (Table 1). The net increase in microbial counts at 24 h was in the order MRS broth (6.2 log cfu/mL) > Eyera (5.1 log cfu/mL) = SESO 3 (5.1 log cfu/mL) > SESO 1 = (4.8 log cfu/mL) > Epuripur (4.9 log cfu/mL).

Values are means ± standard deviations. Values in the same column with similar superscripts are not significantly different (*p* > 0.05). *X*_0_ = Initial microbial count, *X*_max_ = maximum microbial count, and *μ*_max_ _=_ growth rate constant.

The maximal cell concentration of lactobacilli reported in a static pH (6.5) bioreactor using a corn steep liquor medium is 10 log cfu/mL [29]. This is equivalent to the maximal growth observed in this study for MRS broth. The significantly high microbial counts in MRS broth, especially after 18 h, could be explained by the fact that MRS broth is specially developed for the luxurious growth of fastidious *Lactobacillus* spp. [9]. The broth is buffered and highly enriched with nitrogen sources; bacteriological peptone (10 g/L), yeast extract (4 g/L), and beef extract (8 g/L) are important for biomass synthesis [30, 31]. The maximal microbial growth observed in sorghum malt extracts in this study agrees with values reported by Muyanja et al. [19] and Mukisa [23]. These authors reported the ability of *L. plantarum* to grow to 9 log cfu/mL after 24 h of fermentation. The maximum growth observed in the malt extract was also in agreement with Saman et al. [32], who used rice media to grow *L. plantarum* NCIMB 8826. Growth of L. *plantarum* MNC 21 to a lower concentration than observed for MRS broth was probably due to toxicity from lactic acid accumulation [29, 33]. Therefore, maximal microbial biomass growth in sorghum malt extracts could be increased by addition of buffers.

**Figure 2:**
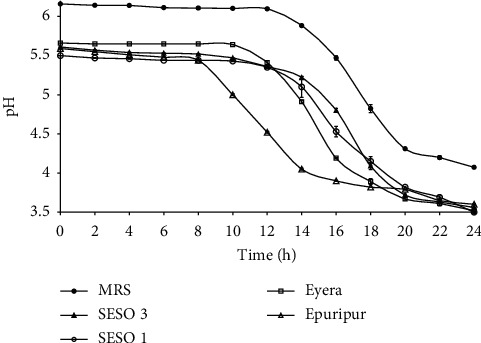
Free amino nitrogen utilization by *L. plantarum* MNC 21 in MRS broth and malt extracts from different sorghum varieties. Error bars show standard deviations of three independent fermentations.

The increase in microbial counts per mg FAN utilized was lower in the sorghum malt extracts (0.2–0.3 log cfu/mg FAN) than in MRS broth (1.5 log cfu/mg FAN). MRS broth has yeast extract, beef extract, and bacteriological peptone which could be more superior nitrogen sources than the sorghum protein. Therefore, the lower (*P* < 0.05) maximum cell counts obtained in the malt extracts could be attributed to the low quality rather than quantity of FAN in the malt extracts since the residual FAN in the broth and malt extracts was still high at the end of the fermentation (Figure 2). Thomas and Ingledew [34] reported a relationship between the quality of FAN and rate of sugar utilization by microorganisms. Higher utilization of sugar by *Saccharomyces cerevisiae* and higher biomass production were reported when the growth medium was supplemented with glutamic acid. However, when replaced with glycine as the major source of FAN, a low sugar utilization and small increase in growth were observed. Therefore, the higher utilization of total sugars in MRS broth could be attributed to the better quality of FAN in MRS broth. Saguir et al. [35] observed glucose utilization and biomass production of *L. plantarum* N4 to be associated with the chain length of amino acids. They noted that addition of dipeptides to growth media increased both sugar utilization and biomass production. Inclusion of free amino acids to the growth media did not increase sugar utilization and biomass production as much as the respective dipeptides. Therefore, growth of microbial biomass in sorghum malt extracts could be improved by selective addition of growth promoting FAN sources.

Although the final cell concentration attained in the sorghum malt extracts was similar, Epuripur would be preferred for biomass production. This is because *L. plantarum* MNC 21 not only exhibited the highest (*P* < 0.05) growth rate but also attained its maximum cell count 6–8 h earlier than the rest of the varieties (Table 1). Statistical analysis indicated varietal differences in the total sugars and FAN levels despite the small ranges. Therefore, the better performance of Epuripur could be attributed to its high endogenous enzyme activity. Epuripur is specifically bred for brewing and possesses high amylolytic activity [36]. It could also possibly have high proteolytic activity resulting in a better nitrogen profile than the other varieties. In a previous study, Epuripur had the highest free amino nitrogen probably resulting from higher proteolytic activity [21].

### 3.2. pH and Acidity

**Figure 3:**
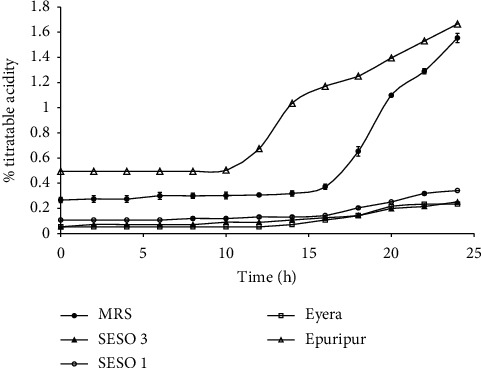
Changes in pH during growth of *L. plantarum* MNC 21 in MRS broth and malt extracts from different sorghum varieties. Error bars show standard deviations of three independent fermentations.

**Figure 4:**
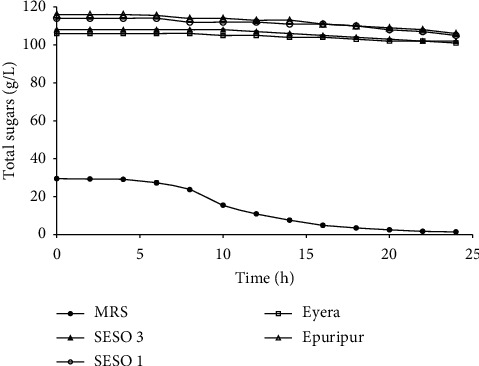
Changes in titratable acidity during growth of *L. plantarum* MNC 21 in MRS broth and malt extracts from different sorghum varieties. Error bars show standard deviations of three independent fermentations.

The trends in pH and TA in the media are shown in Figures 3 and 4, respectively. There was a significant drop in the pH of all media from the start to the end of the fermentation (Figure 3). The pH dropped from 6.2 to 4.1 in MRS broth and from 5.5–5.6 to 3.5–3.6 in the malt extracts. The sharpest pH drop was observed in the first 16–18 h of fermentation. Among the sorghum varieties, the fastest pH drop was in Epuripur which attained pH = 4.0 in approximately 12 h of fermentation. This was followed by Eyera, SESO 1, SESO 3, and MRS broth. The increases in TA were concomitant with the pH reductions (Figures 3 and 4). The highest increase in TA was 1.3% in MRS broth, followed by 1.2% in Epuripur and 0.2% in the rest of the varieties. Rapid acid production was observed between 12 and 24 h of fermentation.

The changes in pH observed in this study are in agreement with values reported for different acid-fermented foods: sorghum and millet [19, 23]), rice [32], wheat [37], and cassava [38, 39]. The pH drop results from conversion of sugars to lactic acid by *L. plantarum* MNC 21. The exponential growth phase (Figure 1) coincided with the sharp drop in pH and a corresponding sharp increase in %TA (Figure 4). Although the highest cell production was observed in MRS broth (Figure 1), this broth had the highest pH (Figure 3). This is attributed to potassium phosphate which buffers the MRS broth [40]. On the other hand, the buffering system in sorghum contributed by protein and ash is relatively weak [41]. Charalampopoulos et al. [8], in a similar study, proposed supplementation of barley malt medium with additives that enhance its buffering capacity so as to reduce acidification rates and increase fermentation times. This is especially important that when microbial growth is done in a batch system, alternatively a continuous system should be used. The estimated lowest pH for the growth of *L. plantarum* is 3.4 [8, 42]. Indeed, the growth rate of *L. plantarum* MNC 21 kept dropping as pH approached 3.4. Extreme low pH conditions cause the undissociated organic acids to penetrate microbial cells and dissociate within the cytoplasm. This alters cytoplasmic pH, hampers normal cellular function, and eventually causes death [43]. Among the varieties, Epuripur had the biggest increase in TA coupled with the highest pH at the end of the fermentation (Figures 3 and 4) possibly implying that it had the best buffering system. Of all the four sorghum varieties used in this study Epuripur in fact has the highest FAN levels [21] suggesting that it could have the highest buffering capacity.

## 4. Total Sugars and FAN

**Figure 5:**
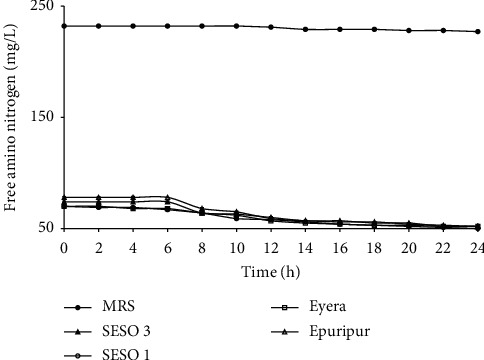
Reduction in total sugars during growth of *L. plantarum* MNC 21 in MRS broth and malt extracts from different sorghum varieties. Error bars show standard deviations of three independent fermentations.

The changes in total sugars (calculated as glucose equivalents) and FAN in the media are shown in Figures 2 and 5, respectively. Initial total sugars in MRS broth were about five times lower than in the malt extracts. A sharp decrease in total sugars occurred after about 8 h of fermentation. The overall decrease in total sugars was 95.3% in MRS broth and 5.0–9.1% in the malt extracts. The rate of sugar utilization in MRS broth was higher than in the malt extracts (Table 2). The initial FAN levels of the malt extracts were about three times lower than those in MRS broth. FAN in MRS broth and the sorghum malt extracts decreased by 2.1% and 24.9–32.7%, respectively. The largest decrease in FAN in the malt extracts occurred in the first 10 h of fermentation. The rate of decrease in FAN was higher in the malt extracts than in MRS broth (Table 2).

Values are means ± standard deviations. Values in the same column with similar superscript letters are not significantly different (*P* > 0.05).

During growth, *L. plantarum* metabolizes glucose to produce primarily lactic acid, and the energy produced is largely used for cell division and maintenance [43]. This conversion results in depletion of sugars with a subsequent increase in %TA. In this study, the malt extracts had much higher sugar levels than MRS broth, but the former had a smaller overall increase (0.2%) in % TA than MRS broth (1.3%). This is may be due to lower sugar utilization in the malt extracts than in MRS (Table 2). Despite the low initial total sugars levels in MRS broth, it produced the highest (*P* < 0.05) cell counts by the end of the fermentation (Figure 1). Therefore, the total sugars in the malt extracts are far in excess and would not be a major limiting factor for growth of *L. plantarum* MNC 21 in the extracts is not.

Although the final microbial count in the extracts was about 9 log cfu/mL, one log lower (*P* < 0.05) than that attained in MRS broth (Figure 1), there was less FAN utilization in the MRS broth than in the malt extracts (Table 2). Unlike the malt extracts, MRS broth contains yeast extract, bacteriological peptone, and beef extracts which are rich nitrogen sources. Therefore, very little of these rich sources of FAN in the MRS are required to cause a significant increase in microbial growth. MRS was originally designed to support growth of the most fastidious LAB [9]. Therefore, it may contain excess nutrients when used for less nutrient demanding species such as some lactobacilli.

This study showed that sorghum malt extracts can be used as alternative growth medium for propagation of *L. plantarum* MNC 21 and possibly other microbial cultures. The carbon and nitrogen sources in the sorghum malt extracts are present in amounts sufficient to support growth of *L. plantarum* MNC 21. Performance of the sorghum malt extracts could be improved by addition of buffers to minimize the toxicity resulting from lactic acid accumulation.

## Figures and Tables

**Table 1:** *L. plantarum* MNC 21 growth parameters in MRS broth and sorghum malt extracts.

Growth medium	*X*_0_ (log cfu/mL)	*X*_max_ (log cfu/mL)	Time (*h*) at *X*_max_	*μ*_max_ (log cfu/ml/h)
MRS broth	3.8^bc^ ± 0.0	10.0^a^ ± 0.0	22	0.8^b^ ± 0.0
SESO 1	4.1^bc^ ± 0.1	8.9^b^ ± 0.0	22	0.8^b^ ± 0.0
SESO 3	3.9^b^ ± 0.1	9.0^b^ ± 0.0	24	0.7^c^ ± 0.0
Epuripur	4.1^a^ ± 0.0	9.0^b^ ± 0.0	16	0.9^a^ ± 0.0
Eyera	3.8^c^ ± 0.0	8.9^b^ ± 0.0	24	0.7^d^ ± 0.0

**Table 2:** Overall utilization of free amino nitrogen and total sugars by *L. plantarum* MNC 21 after 24 h (30°C) in MRS broth and malt extracts from different sorghum varieties.

Media	Rates of decrease in log phase		Overall utilization (%)	
	Free amino nitrogen (mg/L/h)	Total sugars (g/L/h)	Free amino nitrogen	Total sugars
MRS broth	0.3^b^ ± 0.1	1.5^a^ ± 0.1	2.1^d^ ± 0.4	95.3^a^ ± 0.7
SESO 1	1.1^a^ ± 0.1	0.3^b^ ± 0.0	29.4^b^ ± 1.2	8.2^b^ ± 0.3
SESO 3	1.1^a^ ± 0.0	0.3^b^ ± 0.1	31.2^a^ ± 0.8	5.6^c^ ± 0.7
Epuripur	1.4^a^ ± 0.0	0.3^b^ ± 0.0	32.7^a^ ± 0.6	9.1^b^ ± 0.1
Eyera	0.9^a^ ± 0.0	0.3^b^ ± 0.1	24.9^c^ ± 1.1	5.0^c^ ± 0.5

## Data Availability

The data in tables and figures used to support the findings of this study are included within the article.
